# Post-perovskite Transition in Anti-structure

**DOI:** 10.1038/srep37896

**Published:** 2016-11-30

**Authors:** Bosen Wang, Kenya Ohgushi

**Affiliations:** 1 Institute for Solid State Physics, University of Tokyo, Kashiwanoha 5-1-5, Kashiwa, Chiba, 277-8581, Japan; 2 Department of Physics, Tohoku University, 6–3 Aramaki, Aoba, Sendai, 980-8578, Japan

## Abstract

The discovery of the post-perovskite transition, which is the structural transition from the perovskite to post-perovskite structure in MgSiO_3_ under pressure, has aroused great interests in geosciences. Despite of previous extensive studies, key factors of the post-perovsktie transition are still under hot debate primarily due to the big difficulty in performing systematic experiments under extreme conditions. Hence, search for new materials showing the post-perovskite transition under ambient pressure has been highly expected. We here report a new-type of materials Cr_3_*AX (A* = Ga, Ge; *X* = C, N), which exhibits the post-perovskite transition as a function of “chemical pressure” at ambient physical pressure. The detailed structural analysis indicates that the tolerance factor, which is the measure of the ionic radius mismatch, plays the key role in the post-perovskite transition. Moreover, we found a tetragonal perovskite structure with loss of inversion symmetry between the cubic perovskite and orthorhombic post-perovskite structures. This finding stimulates a search for a ferroelectric state in MgSiO_3_.

The discovery of the post-perovskite phase transition, which is the structural transition from the perovskite (pv) to post-perovskite (ppv) structure on the application of pressure in MgSiO_3_, has aroused great interests in geosciences, because this transition can well explain the discontinuity in seismic wave velocities at the deeper mantle around the D” layer^1^,^2^,^3^,^4^,^5^. Since this discovery, various researches have been conducted for the ppv phase of MgSiO_3_; however, a simple question what is the driving force of the ppv transition has not yet be revealed^6^,^7^,^8^,^9^,^10^. The biggest difficulty in elucidating the mechanism is that the ppv phase of MgSiO_3_ is stable only under extreme high pressure of ~120 GPa and unquenchable to ambient pressure^1^,^2^,^3^,^4^,^5^. Thus, the related materials such as CaIrO_3_, CaRuO_3_, and NaMnF_3_, which exhibit the ppv transition under rather lower pressures, have been investigated^7^,^8^,^9^,^10^,^11^,^12^,^13^,^14^,^15^. However, the key factor of the ppv transition is still under hot debate.

We here demonstrate the ppv transition in anti-structure. The anti-structure is the structure where the positions occupied by the anions and cations are opposite to those in the original structure. We focus on a series of compounds *M*_3_*AX (M* = transition metal elements, *A* = main group elements, and *X* = C, N), which possess either anti-pv or anti-ppv structure, which are shown in Fig. 1(a–e), depending on the atomic species^16^,^17^,^18^,^19^. In these compounds, the Cr cations occupy the O^2−^ sites in MgSiO_3_, and the *A* and *X* anions occupy the Mg^2+^ and Si^4+^ sites in MgSiO_3_, respectively. Even though these compounds have been investigated so far with special focus on novel electronic properties, these are also considered to be nice platforms to study the crystal chemistry concerning the ppv transition^18^,^19^. In this paper, we report the details of the structural data for two solid solutions Cr_3_Ga_1−*x*_Ge_*x*_N and Cr_3_GeN_1−*y*_C_*y*_, and give several insights on the ppv transition.

We first summarize the crystal structures involved in this study. The ideal anti-pv structure has the cubic *Pm*-3*m* symmetry. The anti-pv structure is frequently distorted by the mismatch of the ionic radius between the cations and the anions. Assuming that the distortion is induced by the tilted *XM*_6_ octahedra in the rigid corner-sharing framework, we can mathematically derive 15 structures with the supergroup-subgroup relationship shown in Fig. 1(f)^20^,^21^,^22^. Among them, the crystal structures  involved in this study are the cubic *Pm*-3*m* structure with the unit cell of *a* × *a* × *a (a*^0^*a*^0^*a*^0^ tilting) shown in Fig. 1(a), the tetragonal *P*4/*mbm* structure with the unit cell of ~
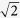
*a *× 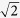
*a *× *a (a*^0^*a*^0^*c*^+^ tilting) shown in Fig. 1(c), and the tetragonal *I*4/*mcm* structure with the unit cell of ~
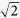
*a* × 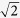
*a* × 2*a (a*^0^*a*^0^*c*^−^ tilting) shown in Fig. 1(d)^23^. Whereas these 15 anti-pv structures are centrosymmetric, there can be also non-centrosymmetrically distorted anti-pv structures. One of them is the tetragonal *P*-42_1_*m* structure with the unit cell of ~
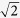
*a* × 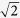
*a* × *a* shown in Fig. 1(e). In this structure, the *XM*_6_ octahedra are seemingly tilted in a similar manner as in the *P*4/*mbm* structure; however, in addition, the *M* atoms are shifted up and down along the *c* axis to form the elongated *XM*_6_ octahedra, resulting in the breakdown of the inversion symmetry. We note that this structure does not belong to the supergroup-subgroup relationship shown in Fig. 1(f). The anti-ppv structure possesses the two-dimensional character with the orthorhombic *Cmcm* symmetry as illustrated in Fig. 1(b), which differs from three-dimensional character of the anti-pv structure.

## Results

We present x-ray diffraction (XRD) patterns taken at room temperature for two solid solutions Cr_3_Ga_1−*x*_Ge_*x*_N and Cr_3_GeN_1−*y*_C_*y*_ in Fig. 2(a). The XRD patterns can be divided into three regions: I. 0 ≤ *x* < 0.50 in Cr_3_Ga_1−*x*_Ge_*x*_N; II. 0.50 ≤ *x* ≤ 1 in Cr_3_Ga_1−*x*_Ge_*x*_N and 0 ≤ *y* < 0.20 in Cr_3_GeN_1−*y*_C_*y*_; and III. 0.20 ≤ *y* ≤ 1 in Cr_3_GeN_1−*y*_C_*y*_. The XRD patterns in region I can be well fitted by assuming the anti-pv structure with the cubic *P*m*-*3*m* symmetry, confirming an earlier report on Cr_3_GaN^23^. In region II, a clear splitting of 0 0 2 reflection in the *a* × *a* × *a* notation into 0 0 2 and 2 2 0 reflections in the ~
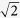
*a* × 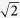
*a* × *a* notation is observed, indicating the symmetry lowering from the cubic to tetragonal one. In addition, there appears new reflections including 1 2 0 and 1 2 1 in the ~
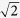
*a* × 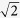
*a* × *a* notations; and all of these reflections are well assigned by the *P-*42_1_*m* space group in consistent with an earlier report on Cr_3_GeN^24^. In region III, the XRD patterns are different completely. All the peaks are well indexed by assuming the anti-ppv structure with the *Cmcm* space group, confirming an earlier report on Cr_3_GeC^25^. We can therefore see that the anti-pv structure in Cr_3_GaN changes to anti-ppv structure in Cr_3_GeC by the chemical substitution or the “chemical pressure”. This transition can be called as the post-pv transition in the anti-structure.

The crystal structures are refined by the Rietveld analysis as shown in Fig. S2, and the volume per unit formula (*V*), the lattice parameters (*a, b*, and *c*), and the bond distances are summarized in Fig. 2(b–e) and Table S1. With increasing *x* in Cr_3_Ga_1−*x*_Ge_*x*_N, the unit cell volume shrinks monotonously in both of the *Pm-*3*m* and *P-*42_1_*m* phases owing to the smaller atomic radius of Ge than that of Ga. On the other hands, with increasing *y* in Cr_3_GeN_1−*y*_C_*y*_, the lattice has a general tendency to expand owing to the larger atomic radius of C than that of N. In the latter course, there is a discontinuous volume decrease by ~0.54% across the anti-ppv transition at *y* = 0.20. This indicates that the negative “chemical pressure” triggers the structural transition to the denser anti-ppv phase. The volume changes in a strong anisotropic manner with increasing *x* and *y*: the *a* axis decreases and the *c* axis increases in the *P-*42_1_*m* phase; and the *a* and *c* axes increase and the *b* axis decreases in the *Cmcm* phase.

To understand the ppv transition from the microscopic viewpoint, we focus on the coordination environment around *A* and *X* atoms, and plot the *A*-Cr and *X*-Cr bond distances as a function of *x* and *y* in Fig. 2 (d,e). Across the transition from *Pm-*3*m* to *P-*42_1_*m* in the anti-pv phases, the 6 *X-*Cr bonds in *X*Cr_6_ octahedra of the *Pm-*3*m* phase are split into 2 longer bonds and 4 shorter bonds with large expansion of the averaged distance, resulting in the elongated *X*Cr_6_ octahedara in the *P-*42_1_*m* structure. In the anti-ppv phase with the *Cmcm* symmetry, on the other hand, the *X*Cr_6_ octahedra are compressed; there are 4 longer bonds and 2 shorter bonds, keeping the similar averaged distances to those in the *P-*42_1_*m* phase. Contrastively, the *A*Cr_12_ polyhedron show more drastic change. The 12 *A*-Cr bonds in an *A*Cr_12_ polyhedron of the *Pm-*3*m* structure are split into 5 groups in *P-*42_1_*m* structure and 4 groups in the *Cmcm* structure. Among them, one group including 2 bonds in the *P-*42_1_*m* structure and 4 bonds in the *Cmcm* structure have much longer bond distances than the other bonds. We can therefore say that the coordination numbers of *A* sites decreases from 12 in the *Pm-*3*m* structure to 10 in the *P-*42_1_*m* structure and 8 in the *Cmcm* structure^26^. We here notice that, in the *P-*42_1_*m* structure, the 2 longer bonds are expanding toward one side of *A*Cr_12_ polyhedron as shown in Fig. S1(d), resulting in the local breakdown of the inversion symmetry. This feature is quite distinct from the centrosymmetrically distorted manner of *A*Cr_12_ polyhedron in the *Cmcm* structure as shown in Fig. S1(e).

To investigate the crystal structure at high temperature, we have collected XRD patterns for Cr_3_GeN in the temperature (*T*) ranges of 25–800 °C; the warming process data are shown in Fig. 3(a). The XRD patterns qualitatively change around 150 and 650 °C, indicating the emergence of the successive structural transitions. The XRD patterns can be well fitted by assuming the *P-*42_1_*m* structure for *T* < 150 °C^24^, the *I*4/*mcm* structure for 150 °C < *T* < 650 °C, the *P*4/*mbm* structure for 650 °C < *T* < 750 °C, and the *Pm-*3*m* structure for 750 °C < *T*. The results of XRD refinements are summarized in Fig. 3(b–e), Fig. S3, and Table S2. On warming, the lattice expands monotonously, and the tetragonal distortions characterized by the *a*/*c* ratio become smaller. In this course, the local environments around the *A* and *X* atoms change in a strange manner. The ratio between the shorter and longer *X-*Cr bonds in a *X*Cr_6_ octahedra, which is the measure of the local tetragonal symmetry breakdown, exhibits anomalous sudden decrease across the *P-*42_1_*m* to *I*4/*mcm* transition. Simultaneously, the *A*Cr_12_ polyhedra change their local environment with 2 + 2 + 4 + 2 + 2 *A*-Cr bonds in the *P-*42_1_*m* structure to that with 4 + 4 + 4 bonds in the *I*4/*mcm* structure in a discontinuous manner. We note that the *I*4/*mcm* structure has similar local environment in *X*Cr_6_ octahedra and *A*Cr_12_ polyhedra to that of the *P*4/*mbm* and *Cmcm* structures (Fig. S1(b), S1(c), and S1(e)), indicating the close connection among these three structures. We can therefore conclude that the *P-*42_1_*m* structure is special among all the structures discussed in this study^20^,^21^,^22^.

To construct the precise phase diagram, we performed differential thermal analysis (DTA) and differential scanning calorimetry (DSC) for two solid solutions Cr_3_Ga_1−*x*_Ge_*x*_N and Cr_3_GeN_1−*y*_C_*y*_ as shown in Fig. 4(a,b). In the DTA data on the warming process for Cr_3_GeN, there are three peaks at around 140, 590, and 690 °C, which correspond to the structural transitions observed in the XRD measurements. By changing compositions, the three structural transition temperatures shift systematically. Similar features are also discernible in the DSC data as shown in Fig. 4(b). Based on these data, the structural phase diagram shown in Fig. 5(b) was established. One can see that the crystal structure of Cr_3_*AX* develops from the cubic anti-pv structure to orthorhombic anti-ppv structure with the intermediate tetragonal anti-pv structure, and that the region of the intermediate tetragonal phase becomes narrower at higher temperature.

## Discussions

We now discuss what the key factor of the ppv transition is. The most plausible candidate is the ionic radius mismatch between *A* and *X* ions, which is measured by the tolerance factor 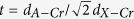, where *d*_*A*−Cr_ and *d*_*X*−Cr_ represents the average atomic distances between the *A* and Cr atoms, and the *X* and Cr atoms, respectively^27^. As shown in Fig. 5(a), the *t* factor calculated by using the atomic radius is 0.94, close to 1 for Cr_3_GaN, indicating the perfect matching of ionic radius and the stable cubic anti-pv structure^28^. With increasing *x* in Cr_3_Ga_1−*x*_Ge_*x*_N and *y* in Cr_3_GeN_1−*y*_C_*y*_, the *t* value becomes much smaller; and then the *A* atoms are not stable in the 12-fold coordination of Cr, destabilizing the anti-pv structure. As a consequence, the anti-ppv structure, in which the *A* atoms have 8 fold coordination of Cr, is stabilized. This is a rough sketch of the ppv transition in the anti-structure.

**Figure 1:**
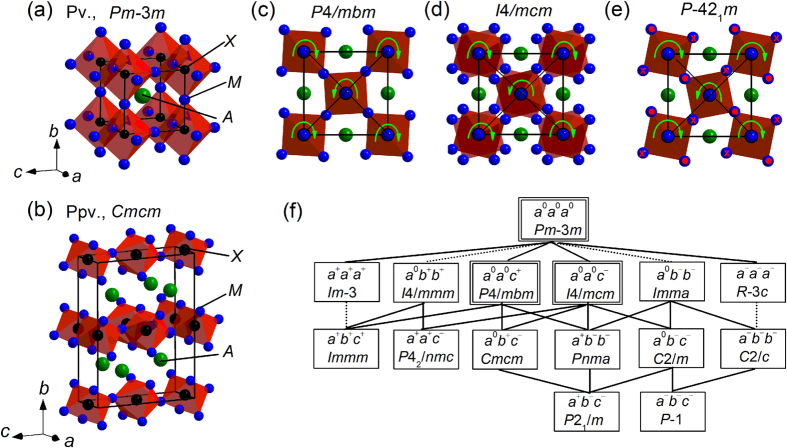
Crystal structures of the anti-perovskite and anti-post-perovskite *M*_3_*AX*. (**a**) The ideal perovskite (pv) structure of the cubic *Pm*-3*m* symmetry, (**b**) the post-perovskite (ppv) structure of the orthorhombic *Cmcm* symmetry, (**c**) the perovskite structure of the tetragonal *P*4/*mbm* symmtery, (**d**) the perovskite structure of the tetragonal *I*4/*mcm* symmetry, and (**e**) the perovskite structure of the tetragonal *P*-42_1_*m* symmetry. (**f**) The supergroup-subgroup relationships among 15 space groups, which are mathematically deduced from the assumption of rigid *X*Cr_6_ octahedra^20^,^21^,^22^. The solid and dotted lines represent the second-order and first-order transitions, respectively. The space group inside the double line is the crystal structure discussed in this study.

**Figure 2:**
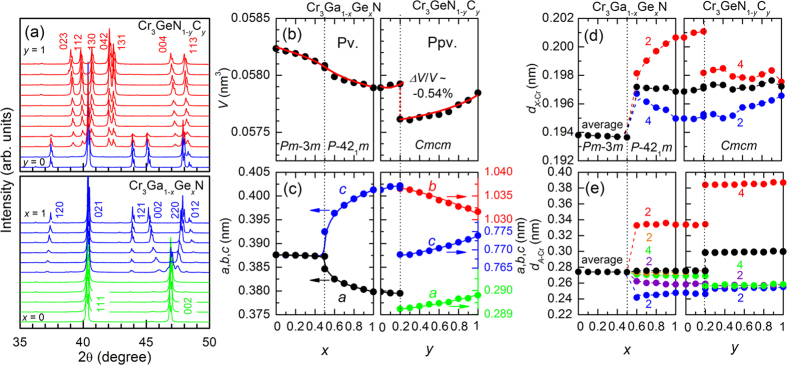
The evolution of the crystal structure at room temperature for Cr_3_Ga_1−*x*_Ge_*x*_N and Cr_3_GeN_1−*y*_C_*y*_ with the composition step of Δ*x* = 0.1 and Δ*y* = 0.1. (**a**) The powder x-ray diffraction patterns in the selected 2θ regions. (b-e) Composition dependence of (**b**) the volume per a formula unit Cr_3_*AX (V*), (**c**) the lattice parameters, (**d**) the bond distance between *X* and Cr atoms (*d*_*X*−Cr_), and (**e**) the bond distance between *A* and Cr atoms (*d*_*A*−Cr_). The number in (**d**,**e**) corresponds to the number of bonds in *X*Cr_6_ ocahedra and *A*Cr_12_ polyhedra.

**Figure 3:**
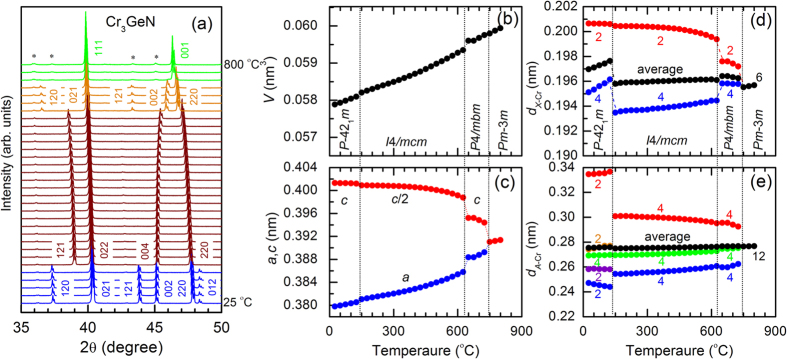
The evolution of the crystal structure at high temperature for Cr_3_GeN. (**a**) The powder x-ray diffraction patterns in the selected 2θ regions at 25–800 °C with the temperature step of 25 °C. Small impurities of Cr_2_O_3_, which appears above 700 °C, are marked by asterisk. (**b**–**e**) Temperature dependence of (**b**) the volume per a formula unit Cr_3_GeN (*V*), (**c**) the lattice parameters, (**d**) the bond distance between *X* and Cr atoms (*d*_*X*−Cr_), and (**e**) the bond distance between *A* and Cr atoms (*d*_*A*−Cr_). The number in (**d**) and (**e**) corresponds to the number of bonds in *X*Cr_6_ ocahedra and *A*Cr_12_ polyhedra.

**Figure 4:**
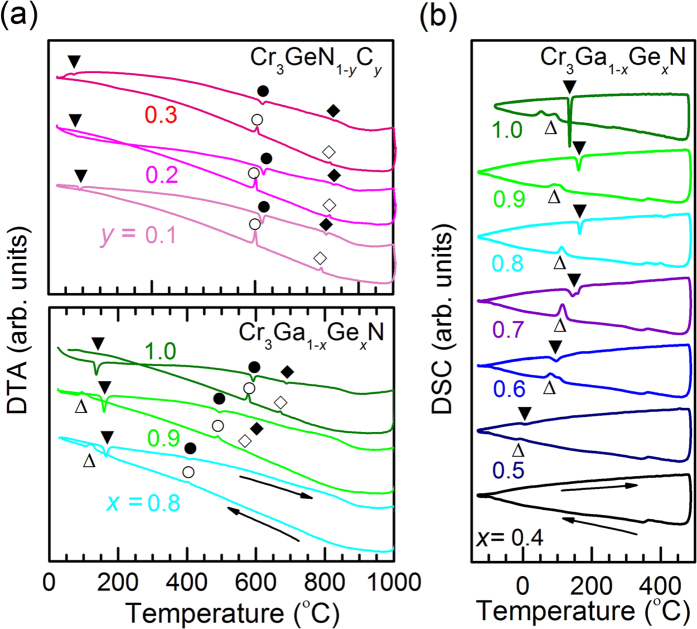
Thermal analysis for Cr_3_Ga_1−*x*_Ge_*x*_N and Cr_3_GeN_1−*y*_C_*y*_. (**a**) The temperature dependence of the differential thermal analysis (DTA). (**b**) The temperature dependence of differential scanning calorimetry (DSC). The data are taken in the warming/cooling processes. Each curve is shifted by the offset for clarity. The symbols indicate the structural transition temperatures.

**Figure 5:**
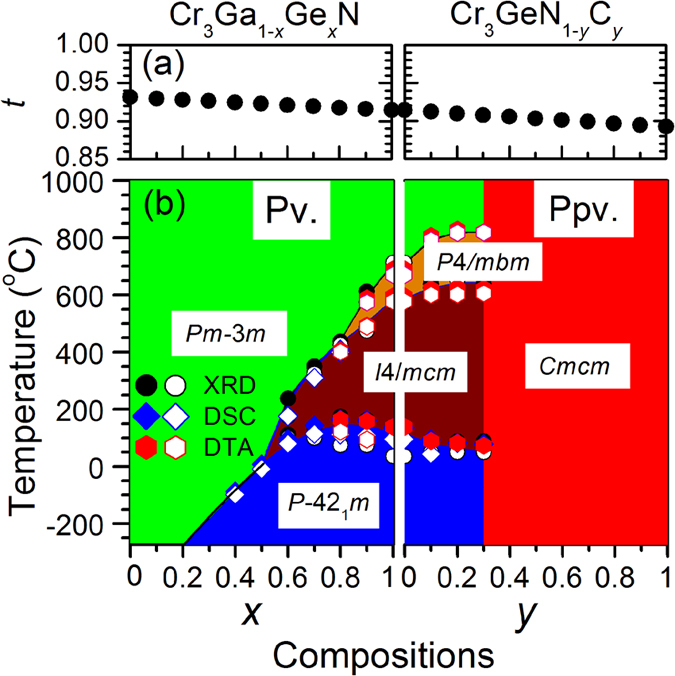
The phase diagram among the temperature-composition plane for Cr_3_Ga_1−*x*_Ge_*x*_N and Cr_3_GeN_1−*y*_C_*y*_. (**a**) Tolerance factor (*t*) estimated by using the atomic radius. (**b**) Phase diagram constructed from the x-ray diffraction patterns, DTA, and DSC. The transition temperature estimated from the data taken on warming and cooling processes are indicated by closed and open symbols, respectively.

In this respect, the three tetragonal anti-pv phases are considered to be the intermediate phases across the ppv transitions. Indeed, the resemblance of the local structure around the *A* atoms among the *I*4/*mcm, P*4/*mbm*, and *Cmcm* structures indicate that the tetragonal distortions in the *I*4/*mcm* and *P*4/*mbm* structures are the precursor of the ppv transition. However, this is not the case for the *P-*42_1_*m* structure. The *P-*42_1_*m* structure is different from the *I*4/*mcm* and *P*4/*mbm* structures in four respects: (1) the structure is not included in the subgroup-supergroup relationship shown in Fig. 1(f); (2) the inversion symmetry is broken; (3) the coordination number of *A* atoms is not 8 nor 12 but 10; and (4) the structural transition temperature is lowered with increasing *y* in Cr_3_GeN_1−*y*_C_*y*_. All of these facts indicate that the *P-*42_1_*m* structure is not stabilized by the ionic radius mismatch between *A* and *X* atoms; instead, the structure is likely stabilized by the covalent nature of N-Cr and Ge-Cr bonds. Such a covalency driven structural phase transitions are discussed in ferroelectrics including PbTiO_3_ and BaTiO_3_^29^, and ferroelectric-like metals including LiOsO_3_, Cd_2_Re_2_O_7_, and Pb_2_Ir_2_O_7_^30^,^31^,^32^,^33^; in both class of materials, inversion symmetry is broken. Therefore, we can conclude that two driving forces of structural modifications, which are the ionic radius mismatch and covalency of chemical bonds, are competing and/or cooperating with each other in the present system. We also notice that the effect of these two driving forces becomes smaller at high-temperature, where thermal fluctuations prefer to high-symmetry structures, which well explains the experimentally observed narrower tetragonal phase at high temperature.

Finally, we discuss the resemblance and difference between the ppv transition in Cr_3_*AX* and that in MgSiO_3_. The ppv transition in Cr_3_*AX* is driven by the ionic radius mismatch between *A* and *X* atoms, which is in harmony with the several proposed scenario of the ppv transition in MgSiO_3_ focusing on the tolerance factor which decreases on the application of pressure^11^,^34^,^35^. Despite of this fundamental resemblance, there are several crucial differences. Firstly, whereas the application of the negative “chemical pressure” induces the ppv transition in Cr_3_*AX*, the application of positive physical pressure induces the ppv transition in MgSiO_3_. This means that the physical pressure is not the key factor of the ppv transition; instead, the tolerance factor is the fundamental factor. Secondly, whereas the pv structure adjacent to the ppv structure belongs to the tetragonal *I*4/*mcm* and *P*4/*mbm* symmetry in Cr_3_*AX*, it belong to the orthorhombic *Pnma* symmetry with the unit cell of ~
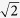
*a* × 2*a* × 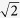
*a (a*^+^*b*^−^*b*^−^ tilting) in MgSiO_3_. Thirdly, not only the structural instability toward to the ppv structure, but also the structural instability toward the inversion-broken state is present in Cr_3_*AX*. We are therefore tempted to imagine that there is a structural instability to the ferroelectric state in MgSiO_3_, in which the Si-O bonds are discussed to have the strong covalent characters^36^,^37^. Further experiments as well as computational simulations are required to clarify this issue. A possible interesting study is a search for new compounds with the anti-ilmenite and anti-LiNbO_3_ structures, which will highlight the important role of the tolerance factor as well as the covalent bonding^38^.

## Methods

Polycrystalline samples were grown by the solid-state reaction. Powders of C (3 N), Cr (3 N), and Cr_2_N (2 N), and grains of Ga (4 N), and Ge (3 N) were mixed in a stoichiometric ratio in a N_2_-filled glove box, and then sealed in a quartz tube under 0.3 atm of Ar gas^18^. The quartz tube was heated to 1000 °C, held for 60 h, and quenched to room temperature. The product was pulverized, and pressed into pellets, which were annealed inside a quartz tube at 1000–1100 °C for 96 h. Then, the above annealing process was repeated. X-ray diffraction experiments were performed by utilizing Smartlab (Rigaku) and M21X (Mac science). Structural parameters were obtained by Rietveld refinement using Rietica software^39^. Differential thermal analysis (DTA) was measured continuously at 25–1000 °C with heating/cooling rates of 20 °C/min. by using the Al_2_O_3_ as the reference. The samples were put into a glass capillary with an inner diameter of 0.1 mm. Differential scanning calorimetry (DSC) was measured at −150–480 °C with heating/cooling rates of 20 °C/min.

## Additional Information

**How to cite this article**: Wang, B. and Ohgushi, K. Post-perovskite Transition in Anti-structure. *Sci. Rep.* **6**, 37896; doi: 10.1038/srep37896 (2016).

**Publisher's note:** Springer Nature remains neutral with regard to jurisdictional claims in published maps and institutional affiliations.

## Supplementary Material

Supplementary Information
