# Supplementary material for: Post-perovskite Transition in Anti-structure
Source: Sci Rep. 2016 Nov 30;6:37896. doi: 10.1038/srep37896 (PMC5129018; doi:10.1038/srep37896)
Supplement: Supplementary Information [file srep37896-s1.pdf]

Supplementary information

Post-perovskite Transition in Anti-structure

Bosen Wang,[1] and Kenya Ohgushi [1,2]

[1]*Institute for Solid State Physics, University of Tokyo, Kashiwanoha 5-1-5, Kashiwa, Chiba 277-8581, Japan*

[2]*Department of Physics, Tohoku University, 6-3 Aramaki, Aoba, Sendai 980-8578, Japan*

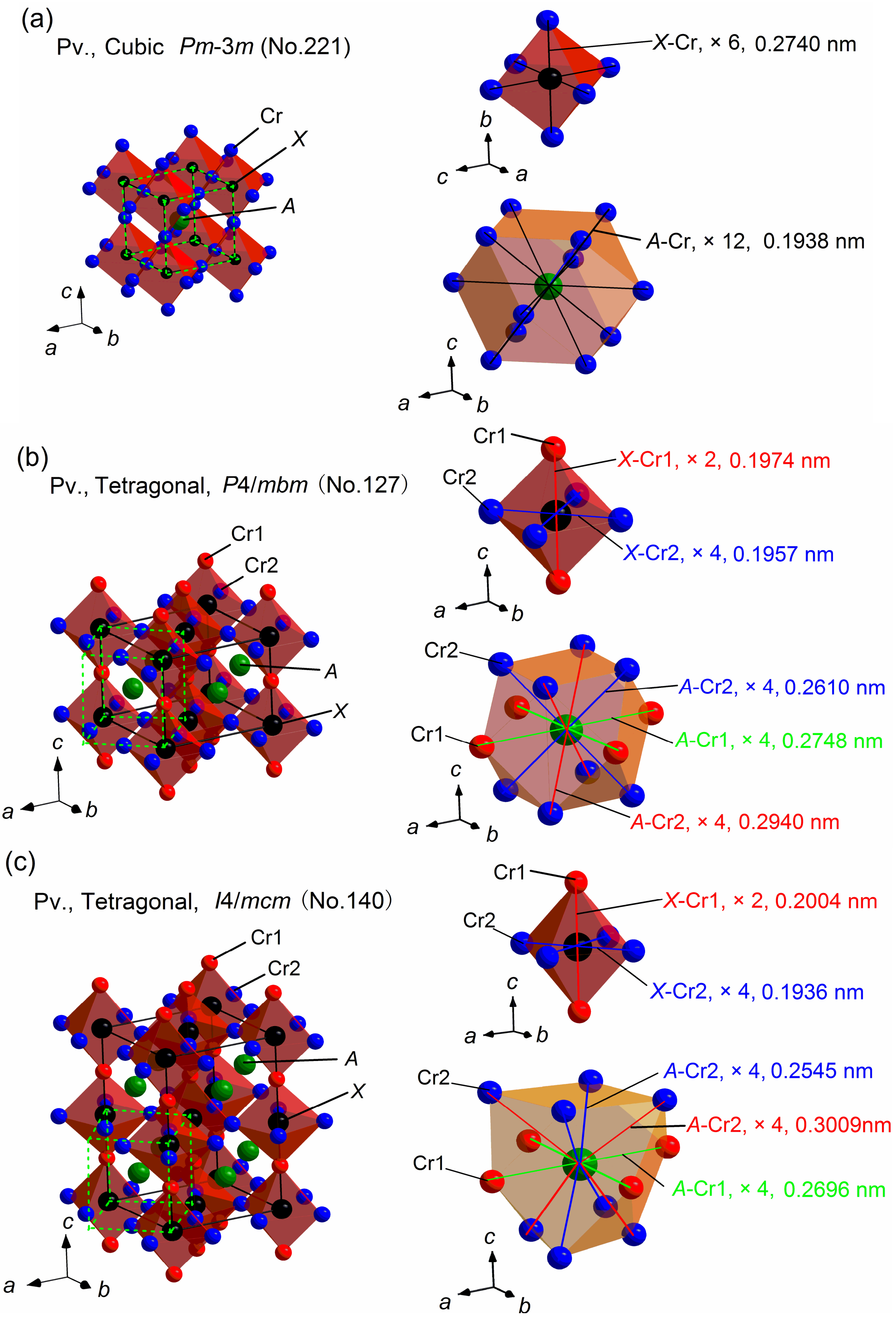
(a)
Pv., Cubic Pm-3m (No.221)
Cr
X
A
c
a
b
X-Cr, × 6, 0.2740 nm
A-Cr, × 12, 0.1938 nm
(b)
Pv., Tetragonal, P4/mbm (No.127)
Cr1
Cr2
A
X
X-Cr1, × 2, 0.1974 nm
X-Cr2, × 4, 0.1957 nm
A-Cr2, × 4, 0.2610 nm
A-Cr1, × 4, 0.2748 nm
A-Cr2, × 4, 0.2940 nm
(c)
Pv., Tetragonal, I4/mcm (No.140)
Cr1
Cr2
A
X
X-Cr1, × 2, 0.2004 nm
X-Cr2, × 4, 0.1936 nm
A-Cr2, × 4, 0.2545 nm
A-Cr2, × 4, 0.3009nm
A-Cr1, × 4, 0.2696 nm

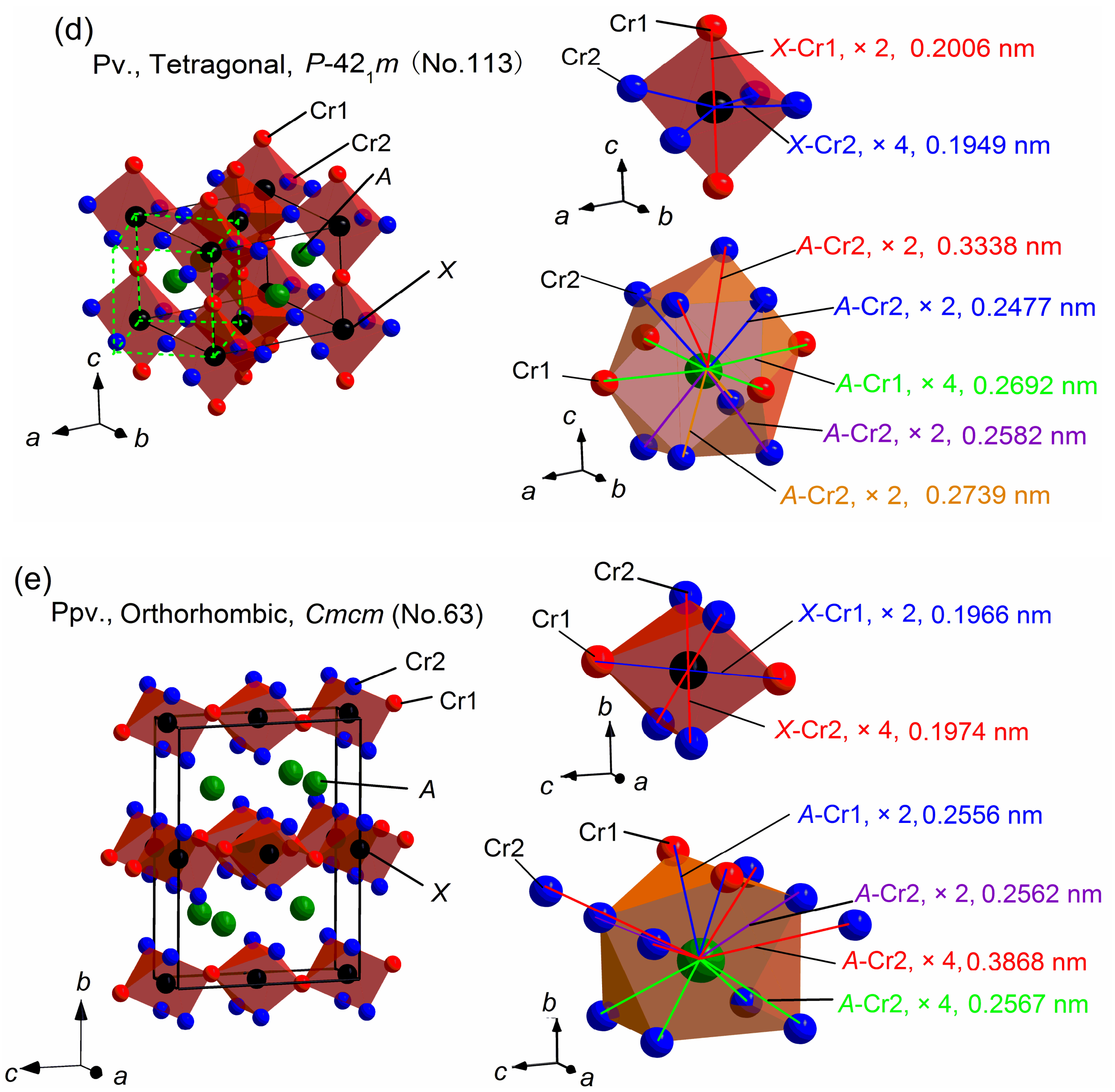

Supplementary Figure 1: Crystal structures of anti-perovskite (anti-pv) and anti-post-perovskite (anti-ppv) compounds, Cr_3AX (*A* = Ga, Ge; *X* = C, N) involved in this study. (a) The cubic anti-perovskite structure with the space group of *Pm*-3*m*, (b) the tetragonal anti-perovskite structure with the space group of *P*4/*mbm*, (c) the tetragonal anti-perovskite structure with the space group of *I*4/*mcm*, (d) the tetragonal anti-perovskite structure with the space group of $P\text{-}42_1m$, and (e) the orthorhombic anti-post-perovskite structure with the space group of *Cmcm*. In the figures, the solid black lines indicates the unit cell, whereas the dashed green lines indicates the unit cell of the original unit cell of undistorted cubic anti-perovskite structure with the space group of *Pm*-3*m*. In the each figure, the coordination environments around the *A* and *X* atoms are also displayed; the bond distances of the *A*-Cr and *X*-Cr bonds with the number of bonds are listed.

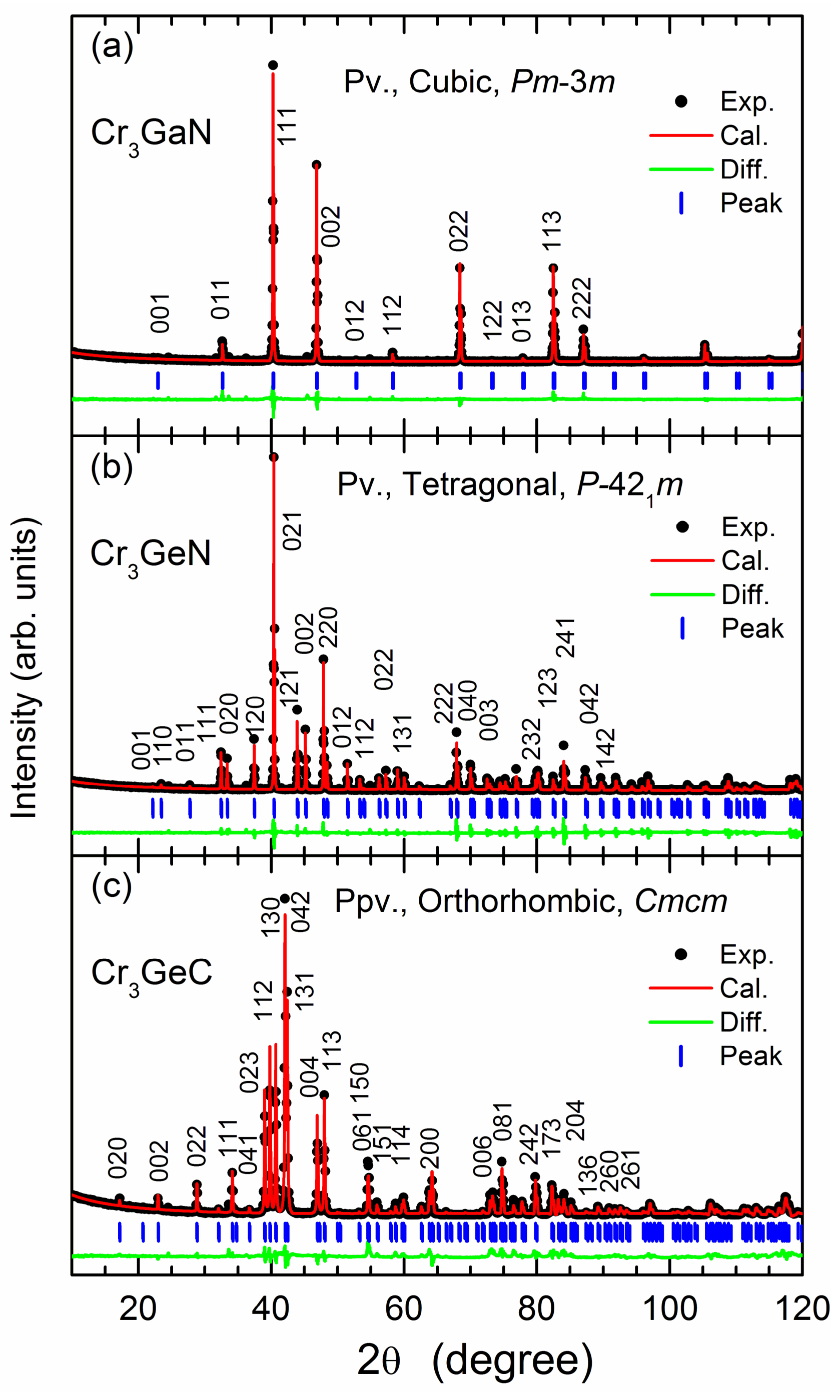

Supplementary Figure 2: X-ray diffraction patterns at the room temperature and results of the Rietveld refinements for (a) Cr_3GaN, (b) Cr_3GeN, and (c) Cr_3GeC. The refined structural parameters are summarized in Table S1.

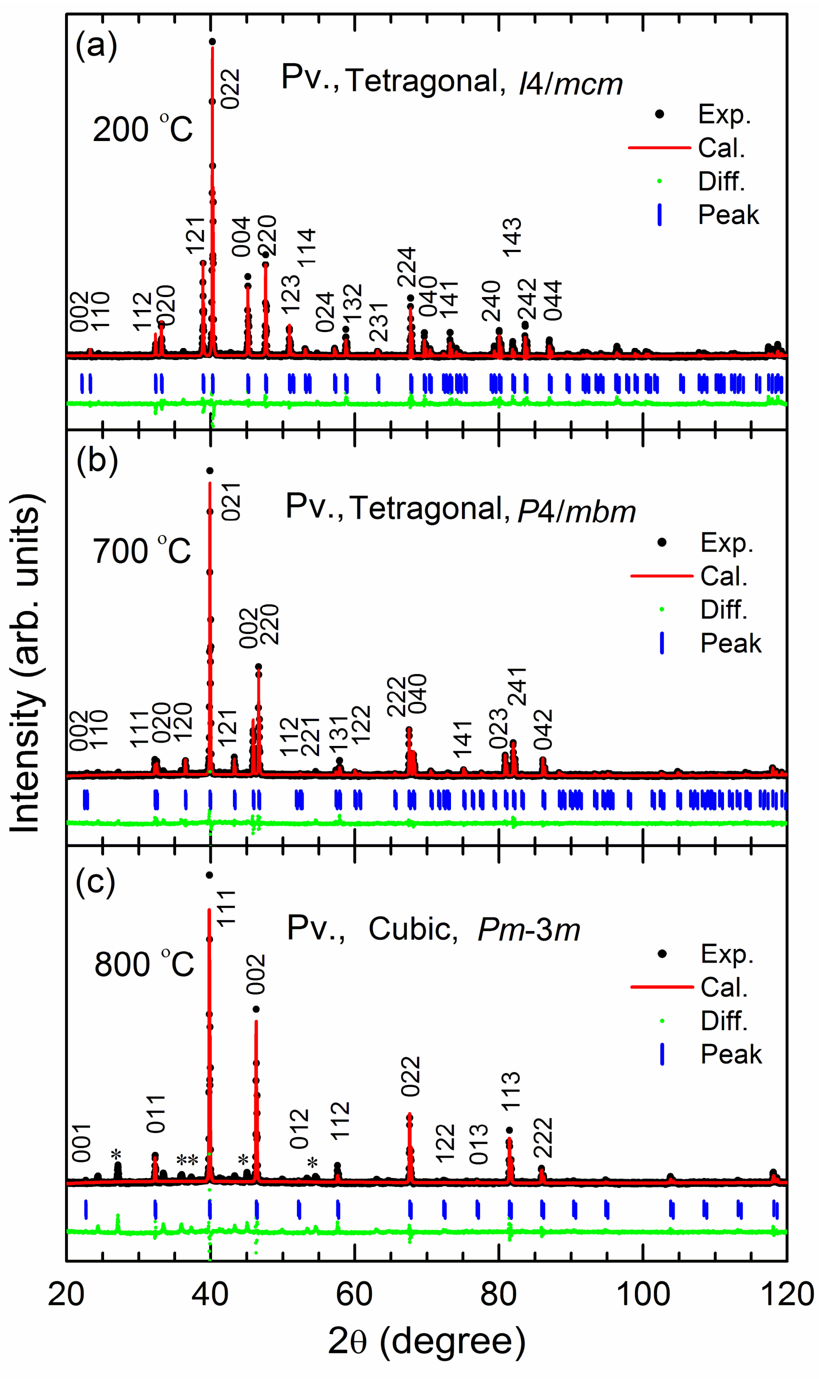

Supplementary Figure 3: X-ray diffraction (XRD) patterns and results of the Rietveld refinements for Cr_3GeN at (a) 200 °C, (b) 700 °C, and (c) 800 °C. The XRD patterns can be well fitted by assuming the *I*4/*mcm* structure at 200 °C, the *P*4/*mbm* structure at 700 °C, and the *Pm*-3*m* structure at 800 °C. The refined crystal parameters are listed in Table S2. At higher temperatures, there are Cr_2O_3 impurities formed by the reaction with oxygen gas.

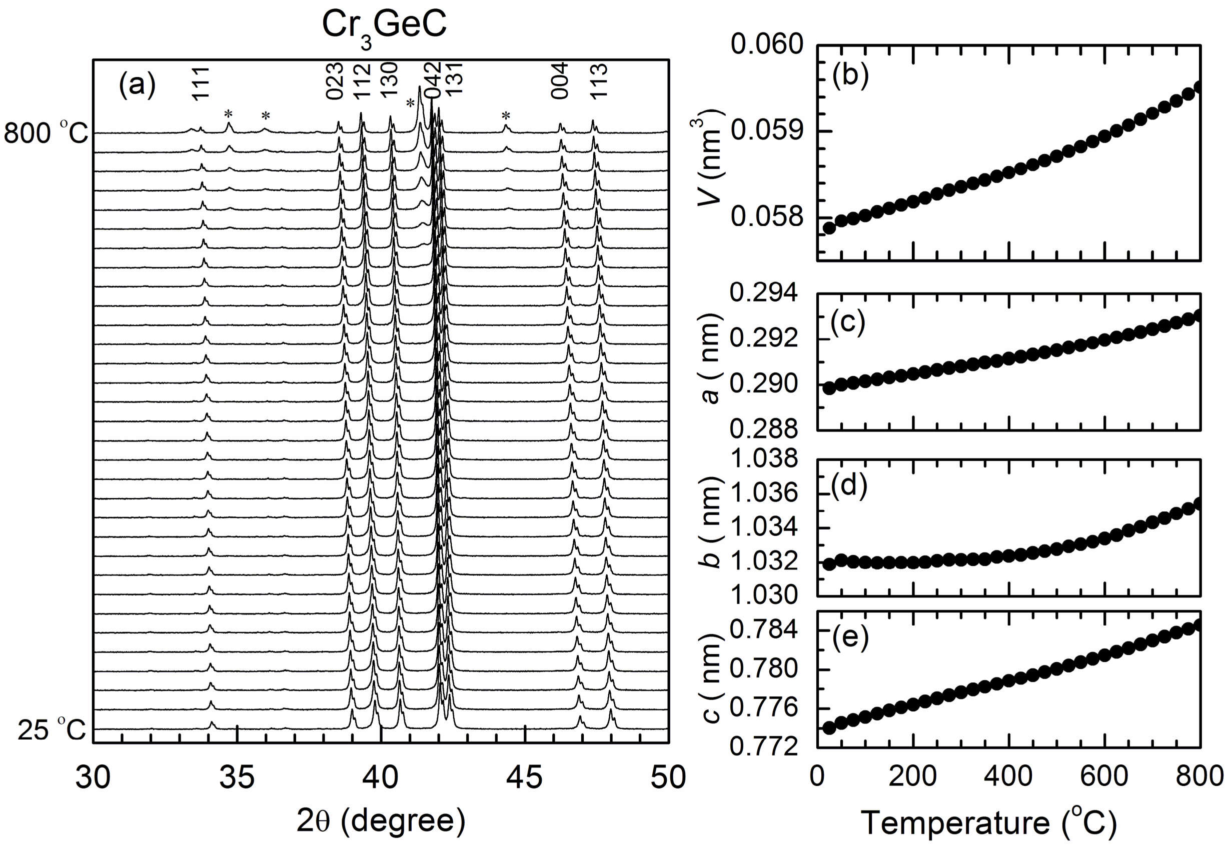

Supplementary Figure 4: X-ray diffraction patterns at high temperature (a), temperature evolution of the obtained volume per a formula unit (*V*) (b), and lattice parameters (*a*, *b*, and *c*) (c-e) for the anti-post-perovskite compound, Cr_3GeC.

Supplementary Tables

Supplementary Table I. Refined Structural Parameters for Cr_3GaN, Cr_3GeN and Cr_3GeC at 25 °C

Composition	Cr_3GaN			Cr_3GeN				Cr_3GeC			
Space group	*Pm*-3*m* (No. 221)			*P*-4$2_1$*m* (No. 113)				*Cmcm* (No. 63)			
Lattice parameters (nm)	*a* = *b* = *c* = 0.38761(1)			*a* = *b* = 0.537064 (3) *c* = 0.401291 (5)				*a* = 0.289806 (3) *b* = 1.031660 (9) *c* = 0.773914 (9)			
z	1			2				4			
Density (10^{-21}g/nm^3)	6.835			6.960				6.906			
Sites	Cr (3*c*)	Ga (1*a*)	N (1*b*)	Cr1 (2*b*)	Cr2 (4*e*)	Ge (2*c*)	N (2*a*)	Cr1 (4*c*)	Cr2 (8*f*)	Ge (4*c*)	C (4*b*)
x	0	0	1/2	0	0.2029(5)	0	0	0	0	0	0
y	1/2	0	1/2	0	0.7029(5)	1/2	0	0.4658(2)	0.1255(1)	0.7617(3)	1/2
z	1/2	0	1/2	1/2	0.0648(5)	0.5482 (8)	0	1/4	0.0450(2)	1/4	0
U_{iso} (10^{-4} nm^2)	0.85(2)	0.77(2)	1.26(21)	0.55(13)	0.71(7)	0.43(9)	0.75	1.337(8)	1.37(7)	0.49(6)	1.512(58)
R_{wp} (%)	1.707			2.013				3.142			
Goodness-of-fit	0.543			0.998				1.235			
Bond distance (nm)	Ga-Cr × 12 0.274082(1)<br>N-Cr × 6 0.193805(1)			Ge-Cr2 × 2 0.2477(5)<br>Ge-Cr2 × 2 0.2582(4)<br>Ge-Cr1 × 4 0.2692(4)<br>Ge-Cr2 × 2 0.27395(1)<br>Ge-Cr2 × 2 0.3338(2)<br>N-Cr2 × 4 0.19497(2)<br>N-Cr1 × 2 0.20065(3)				Ge-Cr1 × 2 0.25560(7)<br>Ge-Cr2 × 2 0.25625(11)<br>Ge-Cr2 × 4 0.25673(3)<br>Ge-Cr2 × 4 0.38686(10)<br>C-Cr1 × 2 0.19667(2)<br>C-Cr2 × 4 0.19742(4)			

Supplementary Table II. Refined Structural Parameters for Cr_3GeN at 200, 700, and 800 °C[1]

Temperature	200 °C				700 °C				800 °C		
Space group	*I*4/*mcm* (No. 140)				*P*4/*mbm* (No. 127)				*Pm*-3*m* (No. 221)		
Lattice parameters (nm)	*a* = *b* = 0.539255(2) *c* = 0.801746 (4)				*a* = *b* = 0.549721(1) *c* = 0.394841 (2)				*a* = *b* = *c* = 0.391354(1)		
z	4				2				1		
Density (10^{-21}g/ nm^3)	6.911				6.752				6.720		
Sites	Cr1 (4*a*)	Cr2 (8*h*)	Ge (4*b*)	N (4*c*)	Cr1 (2*b*)	Cr2 (4g)	Ge (2*c*)	N (2*a*)	Cr (3*c*)	Ge (1*a*)	N (1*b*)
x	0	0.2057(2)	0	0	0	0.2197(2)	0	0	0	0	1/2
y	0	0.7057(2)	1/2	0	0	0.7197(2)	1/2	0	1/2	0	1/2
z	1/4	0.0	1/4	0	1/2	0.0	1/2	0	1/2	0	1/2
U_{iso} (10^{-4}nm^2)	0.75	0.75	0.75	0.75	0.75	0.75	0.75	0.75	0.75	0.75	0.75
R_{wp} (%)	2.969				2.744				3.072		
Goodness-of-fit	1.213				1.247				1.519		
Bond distance (nm)	Ge-Cr2 × 4 0.25450(4)<br>Ge-Cr1 × 4 0.26968(1)<br>Ge-Cr2 × 4 0.30093(5)<br>N-Cr2 × 4 0.19363(5)<br>N-Cr1 × 2 0.200437(1)				Ge-Cr2 × 4 0.261(5)<br>Ge-Cr1 × 4 0.2748(4)<br>Ge-Cr2 × 4 0.2940 (4)<br>N-Cr2 × 4 0.19578 (2)<br>N-Cr1 × 2 0.19742 (3)				Ge-Cr × 12 0.27673(1)<br>N-Cr × 6 0.19568(1)		

[1]The isotropic temperature factor *U*iso was fixed to be 0.75x10^{-4} nm^2 in the Rietvld analysis.
